# Current Approaches to and the Application of Intracytoplasmic Sperm Injection (ICSI) for Avian Genome Editing

**DOI:** 10.3390/genes14030757

**Published:** 2023-03-20

**Authors:** Shusei Mizushima, Tomohiro Sasanami, Tamao Ono, Asato Kuroiwa

**Affiliations:** 1 Faculty of Science, Hokkaido University, Kita 10 Nishi 8, Kita-ku, Sapporo 060-0810, Japan; 2 Faculty of Agriculture, Shizuoka University, 836 Ohya, Shizuoka 422-8529, Japan; 3 Matsumoto Dental University, 1780 Gobara, Hiro-oka, Shiojiri, Nagano 399-0781, Japan

**Keywords:** CRISPR/Cas9, intracytoplasmic sperm injection, primordial germ cells, programmable genome editing, viral infection

## Abstract

Poultry are one of the most valuable resources for human society. They are also recognized as a powerful experimental animal for basic research on embryogenesis. Demands for the supply of low-allergen eggs and bioreactors have increased with the development of programmable genome editing technology. The CRISPR/Cas9 system has recently been used to produce transgenic animals and various animals in the agricultural industry and has also been successfully adopted for the modification of chicken and quail genomes. In this review, we describe the successful establishment of genome-edited lines combined with germline chimera production systems mediated by primordial germ cells and by viral infection in poultry. The avian intracytoplasmic sperm injection (ICSI) system that we previously established and recent advances in ICSI for genome editing are also summarized.

## 1. Introduction

Poultry, such as the chicken (*Gallus domesticus*), turkey (*Meleagris gallopavo*), and Japanese quail (*Coturnix japonica*), are commercially important species because they serve as major food sources worldwide. The poultry industry is indispensable for supporting sustainable development worldwide and is expected to significantly contribute to the United Nations’ Sustainable Development Goals. Studies on the embryos of these birds have contributed to a more detailed understanding of organogenesis [1,2] and human diseases [1], due to their oviparity. Chicken and quail embryos are easy to access and, thus, are amenable to introducing transgenes, normal or transgenic primordial germ cells (PGCs), and viruses [3,4]. The draft genome sequences of chicken, quail, turkey, and zebra finch genomes have been generated, with the information obtained supporting the generation of transgenic birds and the mass production of recombinant proteins [5,6,7,8]. The generation of gene knockout animals is a powerful approach for identifying essential proteins and the functions of uncharacterized genes in many species, as is the case in birds. The combination of recent technical advances is expected to provide novel insights into future aspects of avian biotechnology as well as human society.

The generation of gene knockout chickens by homologous recombination using PGC-mediated methods was initially reported in 2013 [9] following its establishment in mice [10]. Immunoglobulin light chain knockout chickens were subsequently generated using the same method [11]. Although these achievements contributed to the progression of avian gene targeting, the efficiency of this recombination was very low. In this regard, the emergence of site-specific nuclease has provided new avenues for modifications to avian genomes [12,13,14]. The earliest programmable genome editing tools for the generation of gene knockout animals were zinc finger nuclease (ZFN) and transcription activator-like effector nuclease (TALEN) [15]. These enzymes were artificially created by the fusion of FoKI endonucleases with the capacity to recognize long-chain DNA. FoKI endonucleases recognize target DNA and induce double-strand breaks (DSB) and small indels by error-prone non-homologous end joining (NHEJ). An effective TALEN system has been reported in chickens [16,17]. However, the difficulties associated with designing and preparing FoKI endonuclease to function as a fusion protein with a DNA-binding domain, which involves a high level of skill and effort, have hampered the expansion of this technique. To overcome these limitations, the clustered regularly interspaced short palindromic repeats (CRISPR)/Cas9 system was developed as a powerful and convenient tool that has been widely used in various animals, including avian species [12,13,14,18]. In this review, we summarize the application of the avian intracytoplasmic sperm injection (ICSI) system to avian genome editing in addition to current popular approaches.

## 2. CRISPR/Cas9-Mediated Genome Editing Technology

**Figure 1:**
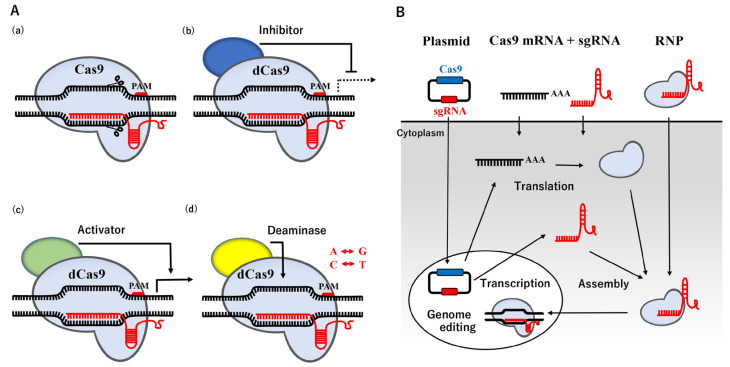
Applications and Routes of the CRISPR-Cas9 system. (**A**) Schematic illustration of the applications. (**a**) A basic molecular model of the CRISPR/Cas9 system. (**b**) dCas9 fused with a transcriptional inhibitor (blue) represses transcription. (**c**) dCas9 fused with a transcriptional activator (green) boosts transcription. (**d**) dCas9 bound to adenine or cytosine deaminase (yellow) modifies A to G or T to C, respectively. (**B**) Schematic illustration of the routes. Three forms of plasmids encoding Cas9 and sgRNA, the RNAs of Cas9 mRNA and sgRNA, or a complex of Cas9 protein and sgRNA are available for delivery into cells by transfection or a microinjection.

The CRISPR/Cas system, originally found in bacteria and archaea, is an RNA-based adaptive immune system that destroys invading plasmids, phages, and viruses [19,20,21]. The nucleoprotein complex, consisting of three crucial components: CRISPR-coding RNA (crRNA), trans-activating crRNA (tracrRNA), and Cas protein, recognize exogenous DNA and degrade it by endonuclease activity [22], causing error-prone NHEJ. The binding site of Cas9 is located upstream of the protospacer adjacent motif (PAM) containing the 5′-NGG base sequence. Humanized Cas9 protein or Cas9 derived from *Streptococcus pyogenes* Cas9 (SpCas9), combined with a synthetic single guide RNA (sgRNA) produced by fusing crRNA with tracrRNA, has been shown to trigger DSB in mammalian cells [23,24]. This CRISPR/Cas9 system may be easily designed and prepared for a plasmid vector by changing the sgRNA sequence to a specific region of the genome sequence instead of ZFN and TALEN described above, and its use has rapidly expanded. The recent re-engineering of Cas9 has resulted in the establishment of dead Cas9 (dCas9) with mutations in two nuclease domains, which has extended the application of the CRISPR/Cas9 system with the potential transcriptional inhibitor/activator, and point mutations [25,26,27,28] (Figure 1A).

Three viral vectors have so far been adopted in clinical trials for the delivery of the CRISPR/Cas9 system: adeno-associated viruses (AAV), adenoviruses, and lentiviruses [29]. AAV are preferred due to their low immunogenicity and stable expression. However, the construction of long Cas9 sequences in plasmids is difficult due to the packaging limitation of AAV. This issue may be partially resolved using truncated SpCas9 [30]. On the other hand, the packaging capacities of adenoviruses and lentiviruses are high. In addition, lentiviruses and adenoviruses show high infection efficiencies in non-dividing cells and non-/dividing cells, respectively. However, lentivirus vectors generally induce insertion mutations through the sustained expression of Cas9 and sgRNA, which may result in off-target effects, while other adenoviruses may induce immune toxicities [31].

In a non-viral method, three forms of the CRISPR/Cas9 system are now available for the delivery of a nucleoprotein complex into the nucleus: plasmid DNA, the RNA system of Cas9 mRNA and sgRNA, and the RNA-protein complex of sgRNA and Cas9 ribonucleoprotein (Cas9 RNP) (Figure 1B). In the plasmid delivery system, the CRISPR/Cas9 plasmid, termed pX330, was originally constructed [23]. The pX330 plasmid contains two expression cassettes, with the expression of sgRNA being driven under the U6 promoter in one and that of Cas9 being driven under the chicken β-actin promotor in the other. In addition, Cas9 is engineered to carry the nuclear localization signal, similar to SV40, which is required to transport the CRISPR/Cas9 system into the nucleus [32]. The RNA system is capable of controllable release into the cytoplasm. The Cas9 RNP system may skip the expression of the Cas9 protein and sgRNA in cellular events, but it also reduces the risk of off-target effects because it avoids overexpression. Although a gene delivery system, such as electroporation and nanoparticle transfer, may be applied to almost any cell type at any stage of the cell cycle, even large-molecule particles, low delivery efficiency remains a challenge due to the large molecular size, instability, and the low efficiency of the endosomal escape [33,34]. Therefore, an alternative method, microinjections, is the most suitable procedure for the Cas9 RNP system, which may directly transfer it to the intended sites, such as the nucleus or cytoplasm, thereby avoiding delivery barriers. The microinjection technique into fertilizing or one-cell stage eggs is generally the most commonly used method for targeted mutagenesis in mammals [35].

## 3. Current Approaches for Avian Genome Editing Based on the CRISPR/Cas9 System

**Figure 2:**
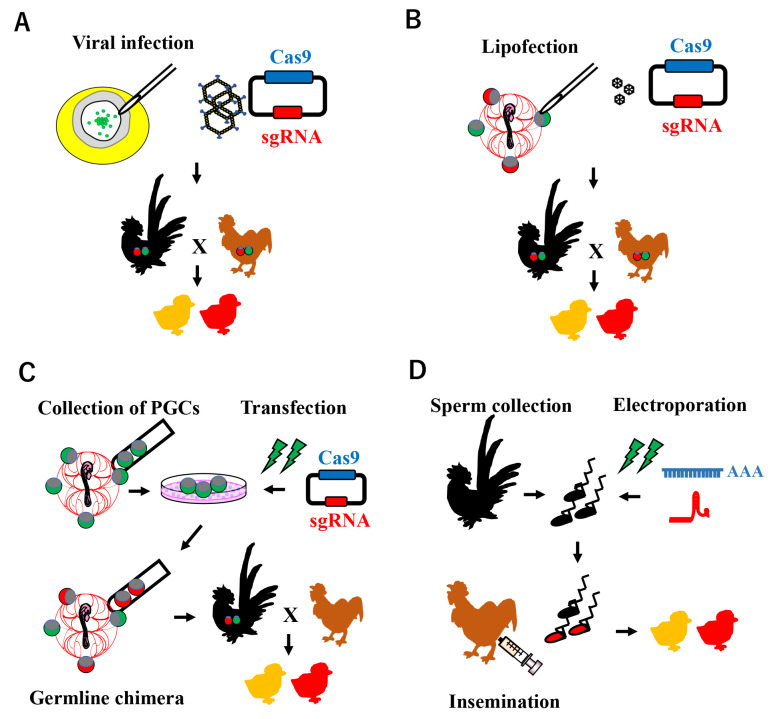
Schematic illustration of CRISPR-Cas9 system-mediated genome editing in poultry. (**A**) Viral infection model: a recombinant adenovirus carrying CRISPR/Cas9 components is infected at blastoderm stage X, where PGCs exist in the center area. (**B**) PGC-mediated genome editing: the plasmid-encoding Cas9 and sgRNA expression cassettes with lipofectamine are microinjected into embryonic blood vessels, in which the PGCs are circulating. (**C**) PGC-mediated genome editing: the CRISPR/Cas9 system is introduced into cultured PGCs in vitro, which are mainly collected from embryonic blood vessels. Enriched genome-edited PGCs are transferred into the blood vessels of recipient embryos to generate a germline chimera. (**D**) Sperm Transfection-Assisted Gene Editing (STAGE): a mixture of Cas9 mRNA and sgRNA is transfected into ejaculated sperm collected from roosters, which are then subjected to artificial insemination in hens.

Avian genome editing technology is based on a procedure for establishing transgenic chicken lines. Various approaches have been attempted to create transgenic birds, including viral infection, the chimeric method, and sperm-mediated gene transfer (Figure 2).

### 3.1. Viral Infection

Viral infection is the most reliable method because of its high efficiency for incorporation of the transgene. The viral gene delivery system was primarily applied to blastoderm stage-X embryos for the purpose of inducing the transgene into the genome of germline cells in the blastoderm [36,37,38,39,40,41,42,43]. Salter et al. [36] was the first to attempt retrovirus transfection into a blastoderm. The infection of a replication-defective pantropic retrovirus vector based on Moloney murine leukemia virus (MoMLV) pseudotyped with the vesicular stomatitis virus G protein was subsequently attempted in Japanese quail stage-X embryos [37]. In that study, the viral vector sequence was detected in the tissue of all the hatched quails, and the efficiency of the germline transmission from G0 to G1 was very high (80%). In addition, the injection of the lentiviral vector derived from the lentivirus equine infectious anemia virus into stage-X embryos was successfully adopted to produce transgenic chickens [38]. Although the expression of the anti-prion single-chain Fv protein was successfully achieved in the eggs of transgenic chickens transfected with the MoMLV-based mouse stem cell virus, the expression level of this protein in the G2 generation decreased to less than that of the G0 founder due to a transgene silencing effect [39]. Therefore, the appropriate selection of key regulator regions for gene silencing or increases in transcriptional activity may be required in future studies that focus on the expression of reporter genes and analyze their functions.

The generation of genome-edited quails was initially achieved using an adenovirus injection containing the CRISPR/Cas9 system targeting the melanophilin (*MLPH*) gene [44] (Figure 2A). Since MLPH functions in feather pigmentation, the feathers of the genome-edited quails were gray. In this study, germline chimeric G0 quails produced genome-edited progeny, and the efficiency of the germline transmission ranged between 2.4 and 10%, suggesting the efficacy of adenovirus infection as a gene delivery system to transduce the CRISPR/Cas9 system for proliferating blastodermal cells. In addition, myostatin gene-edited quails with a higher body weight and muscle mass were successfully produced using the same adenovirus injection [45]. The effects of the adenoviral transduction of the CRISPR/Cas9 system into local tissue were also demonstrated in chick leg muscle [46]. More recently, genome-edited ducklings were successfully produced by an adenoviral infection into blastoderms, indicating the efficacy of genome editing technology with adenoviruses in poultry and water birds other than chickens and quails [47]. Previous studies also confirmed the transduction potential of adenovirus type 5 in chicken, quail, and turkey cells [48,49]. However, this method may not be suitable as a knock-in system because the CRISPR/Cas9 system and donor template must both be delivered into the same cells, indicating low efficiency.

### 3.2. Chimeric Method Using PGCs

Since the viral method may introduce a potentially hazardous infection, non-viral methods for the integration of exogenous DNA into the host genome are preferred. Germ cells are the sole source of the transmission of genetic information to the next generation; therefore, the use of PGCs is currently the most common method for cell-mediated gene transfer because of the higher competency of PGCs than other stem cells, such as embryonic stem cells and embryonic germ cells [50,51,52]. Chicken PGCs appear from the early blastoderm stage and are present as colonies in the central region of the blastoderm at stage X [53,54,55]. After migrating to the germinal crescent region, they circulate in embryonic blood vessels and settle in the embryonic gonads [56]. Due to the unique migratory features of avian PGCs, they may be isolated from various embryonic stages and subjected to long-term cultures without the loss of germ cell potency [57]. Wentworth et al. [58] reported that cultured PGCs settled in recipient gonads after their injection into embryonic blood vessels, resulting in the production of a germ-line chimera in chickens. Isolated gonadal PGCs retained the capacity to migrate and differentiate into mature gametes in the recipient embryo transplanted into their blood vessels [59,60,61].

Using the PGC transplantation method, van de Lavoir et al. [57] reported the successful expression of green fluorescent protein, the reporter gene, in germline chimeras. The transposon system has since been identified as the most efficient for transgenesis [62,63]. Transposon elements have the ability to change their positions within a genome, which mediates the integration of the intended foreign gene. Park and Han reported about 50% germline chimerisms using piggyBac and the Tol2 transposon [63]. Another method involved the direct injection of transposon plasmids into embryonic blood vessels with lipofectamine, with the transformation of the circulating PGCs and the subsequent production of transgenic chickens [64] (Figure 2B). Zhu et al. successfully integrated human immunoglobulin-coded genes in embryonic stem cells derived from stage X [65], indicating the utility of transgenic poultry in industries such as agriculture and biomedicine. 

Genome-editing technologies using the PGC method have been developed in the last decade for basic research on embryogenesis as well as for agriculture and biomedicine. In 2016, two egg-white genes, ovalbumin and ovomucoid, which are well-known allergenic proteins, were efficiently mutagenized in cultured chicken PGCs by the lipofection of the px330 plasmid and an ovomucoid gene-edited germline chimeric rooster was established [66] (Figure 2C). In the same year, transgenic chickens carrying a loxP site in the immunoglobulin heavy chain gene were successfully generated by PGCs using the CRISPR/Cas9-mediated homology-directed repair system (HDR) [67]. HDR is a cellular repair mechanism performed by the homologous recombination pathway that modifies the target genome sequence when an exogenous donor sequence is present at the sgRNA-targeted site in the CRISPR/Cas9 system. A germline chimera carrying a human interferon-β (*hIFN-β*) gene recombined in the ovalbumin locus was then generated by the CRISPR/Cas9-mediated HDR method, and high hIFN-β protein levels were produced in the egg white [68]. Even in basic research fields, a recent study successfully generated a genome-edited chicken targeting doublesex and mab-3-related transcription factor 1 (*DMRT1*) as the testis-determining gene [69].

### 3.3. Sperm-Mediated Genome Editing

Although the chimera production method using PGCs efficiently generates genome-modified birds, a long-term culture of PGCs in vitro is only available in chickens. In addition, two generations are required to reach complete genetically modified birds, including both somatic and germlines. To overcome these drawbacks, a sperm transfection-assisted gene editing (STAGE) method was recently established [70] (Figure 2D). This method involves the direct transfection of spermatozoa with Cas9 mRNA and sgRNA prior to artificial insemination. In this study, genome-edited chicken progeny targeting the *DMRT1* gene locus were successfully produced in one generation; however, the efficiency was low due to multiple factors, such as mRNA stability. Nevertheless, improvements in this protocol may result in its application to a wide variety of avian species, which will provide new research opportunities.

## 4. ICSI-Assisted Genome Editing

The production of gene-edited mice or other animals by the injection of TALEN or CRISPR/Cas9 components into the egg cytoplasm or pronucleus as early as the one-cell stage combined with ICSI or haploid cells is more efficient than that with cell-culture-based strategies [71,72,73,74,75,76,77]. Similar to the avian STAGE, this microinjection technique has the potential to produce genome-edited animals in one generation [78]. In birds, a pronuclear injection is difficult because of the opaque cytoplasm of the egg yolk. However, the major technical limitations hindering the manipulation of avian developmental processes have recently been overcome by the refinement of in vitro fertilization.

### 4.1. Establishment of ICSI

The first avian ICSI system was established with quail eggs in 2003 [79] and was essentially based on the system for avian transgenesis [80,81]. Since the mammalian ICSI system, performed under an inverted microscope, was not applicable to quail eggs because of the large amount of yolk and the opaque cytoplasm, the quail ICSI system was employed using a stereomicroscope. In contrast to mammals (mouse), only a single egg can be recovered from the infundibulum of each female quail, where fertilization occurs in situ, given that it is difficult to induce multiple ovulation [82]. An ejaculated or testicular sperm was microinjected into the center of the germinal disc of a quail egg, and the eggs showed blastoderm development using complete culture techniques developed for one-cell stage eggs to hatching [83]. However, the rate of fertilization at 24 h after ICSI was ~20%, which was low, and they did not develop beyond stage VII even after 72 h of culture [84] because of the difficulties associated with mimicking the physiological polyspermy that occurs during normal fertilization [85].

Unlike mammals, birds exhibit physiological polyspermic fertilization, which is common among reptiles, some amphibians, and most urodeles (newts and salamanders). In poultry, 20 to 60 sperm are generally incorporated into the egg cytoplasm at fertilization [85,86,87,88,89], a number that is markedly higher than that for other polyspermic species [89,90,91,92]. In contrast to monospermic eggs, neither a membrane nor zona pellucida block has been observed in these polyspermic eggs. Only a single sperm nucleus contributes to zygote formation with the female nucleus in a polyspermic egg, with the other sperm nuclei undergoing degradation during early embryo development and thereby ensuring a diploid configuration. However, avian-specific phenomena, such as the movement of supernumerary sperm nuclei towards the peripheral region of the germinal disc before mitosis and their subsequent degradation by maternal deoxyribonucleases, prevent polyploidy [93,94,95,96,97,98]. 

Fertilization in monospermic and polyspermic eggs is accompanied by an increase in the intracellular concentration of Ca^2+^ ([Ca^2+^]*_i_*) immediately after sperm-egg binding or fusion [99,100]. This increase in [Ca^2+^]*_i_* plays a pivotal role in the progression of cell cycle events in animal eggs that are arrested in a species-specific phase of meiosis (metaphase of the second meiotic division in most vertebrate eggs) [99,100,101,102]. The spatiotemporal pattern of the increase in [Ca^2+^]*_i_* associated with egg activation varies widely among species, and we previously revealed a unique pattern of changes in [Ca^2+^]*_i_* in quail eggs after a microinjection of sperm extracts (SE) [103]. This pattern was characterized by an initial transient Ca^2+^ wave followed by long-lasting spiral-like Ca^2+^ oscillations. The transient Ca^2+^ wave was initiated at the injection site of the germinal disc and spread concentrically into the egg cytoplasm. This biphasic pattern of Ca^2+^ signals was only evoked by a microinjection of an SE equivalent into at least 200 sperm but not a single sperm [98]. Furthermore, quail chicks have only been produced by a microinjection of a single sperm together with an SE equivalent into 200 sperm, which suggests that the biphasic pattern of Ca^2+^ signals is essential for fertilization and full-term development to hatching in quails.

In mammalian and newt eggs, phospholipase Czeta1 (*PLCZ1*) and citrate synthase (*CS*) [104,105], respectively, are sufficient to generate the increase in [Ca^2+^]*_i_* required for the initiation of egg activation [84,103,106]. However, aconitate hydratase 2 (ACO2) in addition to PLCZ1 and CS has been identified as a sperm-derived egg-activating factor that is essential for the induction of the biphasic pattern of Ca^2+^ signals in quail eggs [98,103], with PLCZ1 being necessary for the former and CS and ACO2 for the latter. Some quail eggs subjected to ICSI combined with *PLCZ1*, *CS*, and *ACO2* mRNAs underwent normal blastoderm development by 24 h, and hatchlings were obtained from the treated eggs [103,107]. The success of the avian ICSI system opens a new stage of avian genome editing.

### 4.2. ICSI-Assisted Genome Editing

The microinjection technique of foreign DNA into one-cell stage avian eggs was established in the 1980s–1990s, with injected DNA being effectively expressed in the developing embryos. However, DNA expression was gradually lost during embryo development due to the low integration efficiency of the injected DNA into the host chromosome, in contrast to mammals [83,108,109,110]. Since fertilizing chicken and quail eggs obtained by natural mating were supplied for DNA injections in these studies, the presence of supernumerary sperm nuclei was considered to interfere with DNA delivery to the female pronucleus and principal male pronucleus, which fused with the female pronucleus or zygotic nucleus. On the other hand, the greatest advantage of the ICSI technique with a single sperm is that only one pair of male and female pronuclei is formed, thereby reducing the risk of transgenesis and mutagenesis in undesired supernumerary sperm nuclei.

**Figure 3:**
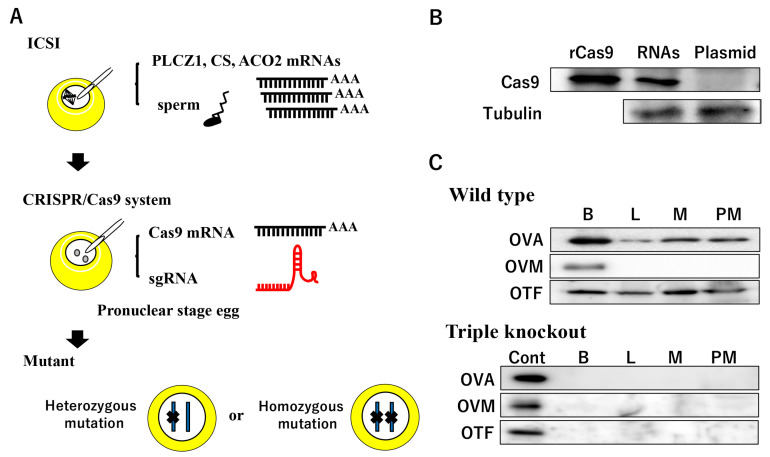
ICSI-assisted genome editing in poultry. (**A**) Schematic illustration of ICSI and administration of the CRISPR/Cas9 system. (**B**) Cas9 protein expression in a one-cell stage egg 1 h after a microinjection of a mixture of Cas9 mRNA and sgRNA or the plasmid encoding Cas9 and sgRNA expression cassettes. Cas9 protein is expressed in the egg by microinjecting RNAs prior to the pX330 plasmid injection. rCas9 is the recombinant Cas9 protein produced by the bacterial expression system. (**C**) Detection of ovalbumin (OVA), ovomucoid (OVM), and ovotransferrin (OTF) proteins in a D14 embryo after a microinjection of Cas9 mRNA and three sgRNAs targeting the OVA, OVM, and OTF gene loci into the one-cell stage of a quail egg. Cont, protein extract of the whole brain in a sham-operated embryo; B, whole brain extracts; L, liver extracts; M, mesonephros extracts; PM, pectoral muscle extracts.

We previously attempted to microinject an RNA mixture of hCas9 mRNA and sgRNA synthesized in vitro into a one-cell stage quail egg 2 h after ICSI, and a cyclin D1 (*CCND1*) locus-targeted quail mutant was obtained (Figure 3A). As expected from the expression pattern in blastoderm development, the *CCND1* mutant blastoderm showed developmental arrest at the predicted stage [111]. Notably, more than 70% of the blastoderms carried the biallelic mutation. These homozygous mutations were also occasionally identified at the 3′-UTR of the *CCND1* locus in all blastoderms, suggesting that the CRISPR/Cas9 system mediated by ICSI has the potential to produce genome-edited birds in one generation. Genome-edited efficiency was compared between the px330 plasmid and synthesized CRISPR/hCas9 RNAs, and the mutagenesis in the plasmid injection was lower than that of RNAs due to the delayed onset of the Cas9 mRNA transcription or subsequent translation (Figure 3B). In addition, our preliminary data revealed that the ICSI-assisted CRISPR/Cas9 system was capable of simultaneous DSB at three different genes in the quail zygote (Figure 3C, unpublished data).

Since the conventional PGC gene targeting strategy is time consuming, involving the cultivation of PGCs, the selection of genome-edited PGCs, and the production of germline chimera and progeny, the current approach to one-cell stage eggs significantly shortens the period needed for the production of mutants in birds. Therefore, we conclude that the current ICSI-mediated genome editing technology is a straightforward and fast approach to generate targeted gene knockout poultry. However, this technique requires considerable technical skill and labor in addition to several instruments used in micromanipulation and microinjection, such as the micromanipulator and microinjection systems. Since the hatching rate is still low, further studies are also warranted.

## 5. Conclusions

Genome editing technology has rapidly advanced in recent years, providing an adaptable tool for the effective manipulation of the genomes of various animals. Although genome editing in avian species has historically been challenging, the emergence of the CRISPR/Cas system has accelerated avian research. Poultry contributes eggs and meat as a major source of protein. They also provide numerous opportunities to produce allergen-reduced eggs and many drugs that are related to human health and diseases in egg whites as a bioreactor for potential pharmaceutical and industrial applications via the CRISPR/Cas9 system. PGC-mediated mutagenesis is currently only available for chickens but is driving the development of alternative technologies, such as adenoviral infection and ICSI in quails. Therefore, avian CRISPR/Cas9 techniques are still in their infancy but are swiftly evolving. In mammalian studies, the nanoparticle-mediated targeting delivery of the CRISPR/Cas9 system to the intended cells or organs has begun to be intensively pursued by many researchers [33,34,112], with their potential application to avian PGCs in the future. Further technical improvements and the development of novel technologies will pave the way for genome editing technology with the desired avian agricultural outcomes. While simple constitutive knockout is useful and beneficial, it should be noted that it is desirable to develop conditional loss-of-function models, especially for genes essential for cell survival and embryonic development.

## Data Availability

Not applicable.
